# Correlation of Temporal Parameters of Laryngeal Excursion by Using Force-Sensing Resistor Sensors with Hyoid Motion in Videofluoroscopic Swallowing Study

**DOI:** 10.1007/s00455-020-10121-2

**Published:** 2020-04-28

**Authors:** Chin-Man Wang, Chao-Jan Wang, Wann-Yun Shieh, Yen-Chia Chen, Wei-Jen Cheng, Wei-Han Chang

**Affiliations:** 1 Department of Physical Medicine and Rehabilitation, Linkou Chang Gung Memorial Hospital, College of Medicine, Chang Gung University, Gueishan District, No.5, Fu-Hsing Street, Taoyuan City, 33305 Taiwan R.O.C.; 2 Department of Medical Image and Intervention, Linkou Chang Gung Memorial Hospital, College of Medicine, Chang Gung University, Taoyuan City, Taiwan R.O.C.; 3 Department of Computer Science and Information Engineering, Chang Gung University, Gueishan District, No. 259, Wen-Hwa 1st Road, Taoyuan City, Taiwan R.O.C.

**Keywords:** Deglutition, Deglutition disorders, Swallowing temporal parameters, FSR, VFSS, Correlation

## Abstract

Small flexible force-sensing resistor (FSR) sensors can detect laryngeal excursion during swallowing, but the detected laryngeal excursion has not been correlated with videofluoroscopic swallowing study (VFSS) results. Here, we tested the correlation of temporal parameters between the laryngeal excursion recording by FSR sensor and the hyoid motion recording by VFSS under simultaneously swallowing test recordings. Swallowing measurements were recorded in a radiological suite by simultaneously using VFSS and FSR sensors to detect hyoid motion and laryngeal excursion, respectively. Volunteers sat with their head vertical to the Frankfort plane. Two FSR sensors, each for detecting thyroid cartilage excursion and thumb pressing, were placed. VFSS images and FSR sensor signals during single 5-mL barium liquid (30% wt/volume %) bolus swallowing were collected and analyzed for four swallows per participant. In total, 15 men (28.0 ± 4.1 years old); 14 women (28.4 ± 4.2 years old) were recruited. Temporal parameters between VFSS and noninvasive system demonstrated a strong correlation by Pearson’s correlation analysis: in men (*R* = 0.953–0.999) and in women (*R* = 0.813–0.982), except for VT1–V1 compared with FT1–F1, which demonstrated a moderate correlation in women (*R* = 0.648; all *p* < 0.001). Only VT1–V1 and FT1–F1 in women displayed a significant difference (*p* = 0.001). Therefore, this is the first study to simultaneous record VFSS and noninvasive signals by FSR sensor. The correlation of temporal parameters between these two tests was strong. This finding is valuable for future applications of this noninvasive swallowing study tool.

## Introduction

Noninvasive swallowing studies, a new trend, have several advantages. Because it does not involve radiation exposure, it is suitable for studying early, subtle, or mild dysphagia [1, 2], and is favorable for collecting large amount of swallowing data, particularly if short term follow-ups and repeated measurements are necessary [3]. Furthermore, noninvasive swallowing measurements could be useful for differentiating normal and pathological swallowing physiology in the future.

Recently, noninvasive oropharyngeal swallowing study tools are being used, and various noninvasive sensors are used to detect and record thyroid cartilage excursion from the skin surface. These sensors include piezoelectric sensors, accelerators, bend sensors, force-sensing resistor (FSR) sensors [1, 4–9], and sonography [10]. Concurrently, a stethoscope with an amplifier or microphone can record and collect the sounds of swallowing [11–13]. Each sensor and method has strengths, but different laboratories have objectives, conditions, and other factors (e.g., being familiar with software or hardware already in their laboratory), which may help them to make a choice between different sensors.

Our team used piezoelectric [7, 9] and FSR [1, 2, 8, 14] sensors to monitor thyroid cartilage during swallowing. However, finding new sensors and comparing their results with those from videofluoroscopic swallowing studies (VFSS) is valuable with regard to research and clinical applications.

Detecting thyroid cartilage excursion is a key feature of noninvasive swallowing study. A study demonstrated that excursion of thyroid cartilage is synchronized with hyoid motion during VFSS [15]. When swallowing responses start, the hyoid bone moves upward and forward promptly, remains at the highest point, and then, returns to its initial position [15–17]. Hyoid bone and thyroid cartilage demonstrate synchronized movement during swallowing, referred to as hyolaryngeal movement [18, 19] or hyolaryngeal excursion [20]. Notably, these sequential movements are related to swallowing respiratory pause and to the timing and duration of upper esophageal sphincter relaxation during swallowing. These timely coordinated movements allow the bolus to pass the cricopharyngeal sphincter smoothly and securely [6, 21]. Therefore, monitoring and analyzing hyoid motion during VFSS is crucial. Monitoring thyroid cartilage excursion in noninvasive swallowing studies using surface sensors is also vital.

The results from noninvasive swallowing studies using FSR sensors to detect the thyroid cartilage excursion, however, were not correlated with those from VFSS. Therefore, our team synchronized these two systems to verify if the temporal parameters between the systems were correlated and postulated that laryngeal excursion detected using FSR sensors is synchronized with the hyoid motion during VFSS. This study aimed to identify the relationship between the temporal swallowing parameters of laryngeal excursion signals detected using FSR sensors and hyoid motion images detected during VFSS, which were recorded simultaneously in young volunteers without dysphagia.

## Materials and Methods

### Volunteers and Ethical Approval

The Ethics Committee of Chang-Gung Memorial Hospital approved the study protocol (No. 201602016B0C101). Each participant was introduced to and thoroughly explained the study’s aims and procedures and asked to sign the informed consent form.

All participants were young and healthy. Exclusion criteria included a history of dysphagia, cardiopulmonary disease, neurological disease, chronic indigestion disorder, diseases of the head and neck, and smoking in the previous 5 years. All volunteers were administered the Functional Oral Intake Scale (FOIS) [22] and Eating Assessment Tool (EAT-10) [23]; the results on neither of the scales demonstrated dysphagia. In total, 29 healthy volunteers [15 men (mean age: 28.0 ± 4.1 years) and 14 women (mean age: 28.4 ± 4.2 years)] were recruited. The participant characteristics are listed in Table 1.

**Table 1:** Characteristics of young healthy volunteers without dysphagia

	M (No:15)	F (No:14)	*P-*vale
Age (year/old)	28.00 ± 4.13	28.43 ± 4.22	0.784
BMI (kg/m^2^)	22.69 ± 4.90	22.48 ± 1.81	0.124
Height (cm)	173.8 ± 6.0	163.4 ± 5.1	0.000
Weight (kgs)	68.60 ± 15.85	54.86 ± 7.37	0.006

*M* male, *F* female, y/o year old, *BMI* body mass index

### Instruments (Hardware and Software)

The ambulatory noninvasive swallowing respiration assessment system based on the MP150 system (MP150 system, Biopac, Goleta, CA, USA) was sent to radiological suites and set up for simultaneous recording by using a VFSS equipment (Luminos; Siemens).

### Biopac System

**Fig. 1:**
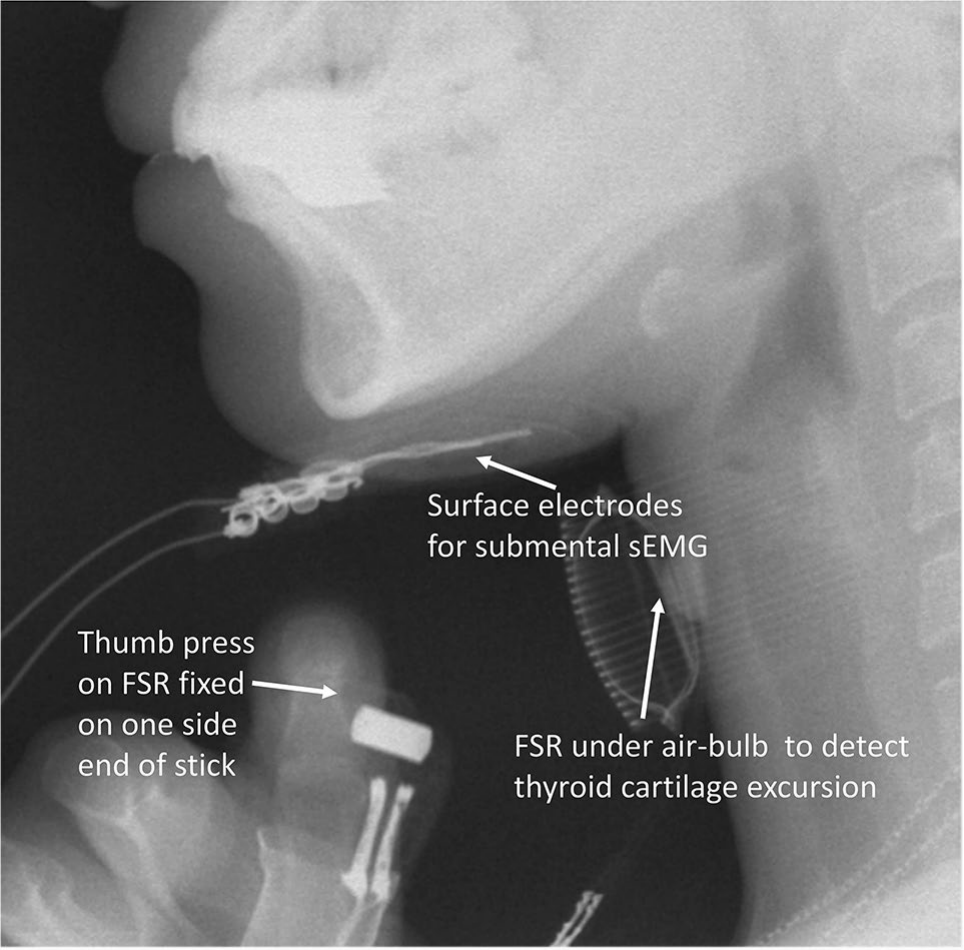
FSR sensor attached to the side of a small handheld stick to detect thumb pressing, and FSR sensor under an air bulb to detect thyroid excursion under VFSS

Two FSR sensors were used—one to detect thumb pressing and the other to detect thyroid cartilage excursion. Noninvasive swallowing and respiration coordination signal monitoring based on the Biopac system was set up in our laboratory when FSR sensors were used to detect thyroid cartilage excursion in previous studies [1, 3, 7–9]. To collect the test data for the correlation of temporal parameters of signals detected using FSR sensors with images recorded in VFSS, a noninvasive swallowing respiration system based on Biopac, including recording of submental sEMG and nasal airflow for respiration, were also recorded for verification. Moreover, one FSR sensor was fixed at the thyroid cartilage level on the midline of the anterior neck skin surface to detect thyroid cartilage excursion [1]. Another FSR sensor was fixed on the upper side of a handheld stick which was designed as an event marker to detect thumb pressing motions (Fig. 1). The swallowing signals using noninvasive systems and images using VFSS were also recorded simultaneously, and the motion of thumb pressing signals were used as time reference points for the two swallowing study systems.

### Vfss

For the VFSS, the recording speed was 30 frames/s in the lateral view and the time duration was 1/30-s interval per image.

#### Simultaneously Recording Of Swallowing-Related Thyroid Cartilage Excursion Signals Using MP150 System And Hyoid Motion During VFSS

To overcome the problem of synchronizing the two systems Biopac system and VFSS, another FSR sensor, on the upper end of a handheld stick designed to detect a thumb pressing, was used. These thumb pressing reference points enabled the analysis of timepoints and durations between the two systems. The noninvasive oropharyngeal swallowing signals, including those detected using FSR sensors, were simultaneously recorded during VFSS and analyzed offline later using LabView (version 2010; Mathworks, Natick, MA, USA).

### Software

Acqknowledge (version 5.0; Biopac) for the MP150 system was used for recording swallowing signals, and LabView was used for the subsequent offline analysis.

### Study Procedures

**Fig. 2:**
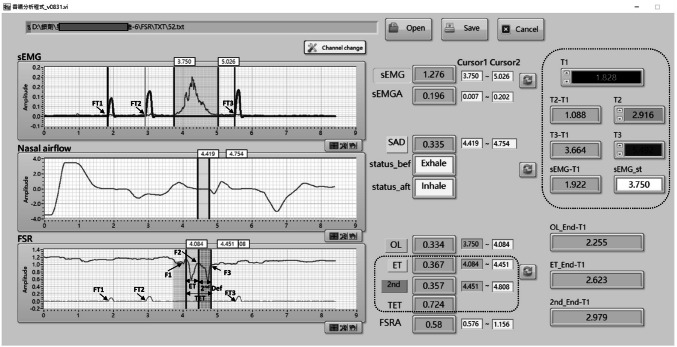
LabView for noninvasive swallowing and respiration parameter analysis and the three events of thumb pressing on FSR sensor

To validate FSR sensor detection of temporal swallowing parameters, an ambulatory noninvasive swallowing study system (based on the MP150 system) was used and a VFSS was performed simultaneously in radiological suites. In this study, a specially designed FSR sensor on one side of a small stick was held in the palm to detect thumb pressings as the reference time events for analysis. Volunteers were equipped with two ECG adhesive electrodes for submental sEMG, a nasal cannula for respiration, an FSR sensor for the laryngeal excursion, and an FSR sensor for thumb pressing—all of which were connected onto the MP150 system. These validation study procedures included an FSR sensor held in the volunteer’s hand, which was pressed with the thumb twice—before swallowing and once after swallowing—and another FSR sensor was fixed onto the anterior neck at thyroid cartilage level to detect thyroid cartilage excursion during 5-mL barium swallowing in VFSS. During VFSS, the volunteers sat on a chair with their head vertical to the Frankfort plane. Few small water boluses at room temperature were dispensed to volunteers for the setting up a noninvasive measurement system and to familiarize the volunteers with the test procedure. Liquid barium (30% wt/volume %; 5 mL) bolus was administered using a 20-mL disposable syringe into the mouth of the volunteers. All participants were instructed to hold the FSR sensor fixed on one side of small stick in their hands and hold the fed 5-mL barium bolus in their mouths; they were then asked to press the FSR sensor and swallow the barium bolus on verbal instruction by the radiologist in the following order: “press” (thumb press the FSR sensor on the stick), “press,” “swallow,” and “press”. In total, four separate single boluses of 5-mL barium were recorded each individually by using the two systems simultaneously. In the lateral view in the VFSS, the dynamic images of oropharyngeal swallowing were recorded with 30 images/s. All VFSS videos were recorded and saved in an picture archiving and communication system (PACS). After the study was completed, the copies of VFSS videos from PACS were exported for later offline analysis. The recorded swallowing and respiration signals were exported for subsequent offline analysis (Fig. 2).

The total radiation exposure time was controlled to < 2 min [4] because the dose of radiation in similar circumstances is approximately one-third of clinical VFSS in patients with dysphagia at our hospital, which is approved by our radiation safety committee and institutional review board. No choking events were noted during the study. No volunteer displayed signs of dysphagia or aspiration during the VFSS combined with noninvasive swallowing study. All the participants exhibited normal tongue base retraction, epiglottis inversion, epiglottis seal, and upper esophageal sphincter (UES) opening during barium bolus VFSS.

#### Defining Time Events (Onset Timepoints) and Durations on VFSS Images and Noninvasive Swallowing Assessment Tool Signals During Swallowing

A single-frame analysis was used in our VFSS image analysis [24]. To calculate the time and duration of hyoid motion [25], the frame number of each image recording was used to calculate the time when the action occurred as well as the onset timepoint and duration of each motion.

**Fig. 3:**
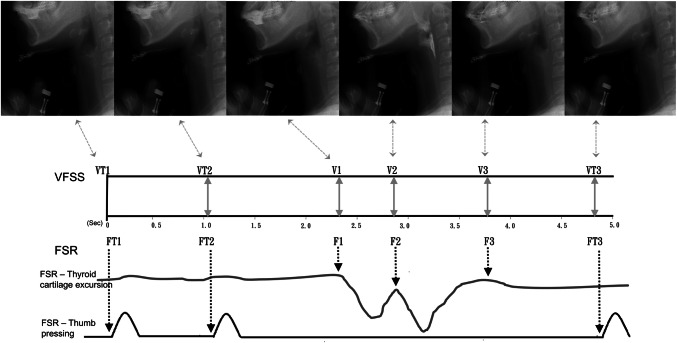
Timepoints on the signal waveform from the FSR sensor (FT1–FT3, F1-F3) and VF events (VT1-VT3, V1–V3)

To accurately analyze hyoid displacement, the investigators underwent 3 months of extensive training on the biomechanical analysis of swallowing and VFSS before joining this investigation. Two independent trained investigators manually selected three target frames from the timeframe set for the FSR sensor and three target frames of hyoid bone-related sequence movement during swallowing including the frame of movement initiation, movement of the hyoid bone toward the frame at uppermost point, and movement back to the resting position. The resting and uppermost frames of the hyoid bone movement during swallowing were selected only after multiple observations of the entire swallow in real-time and after frame-by-frame inspection. The resting position of the hyoid bone was marked as the position that hyoid was in the moment before the bolus was propelled into the pharynx from the oral cavity. After each frame was defined, the time duration between the three targeted swallowing frames and three thumb pressing frames were calculated (Figs. 2 and 3). There were some disagreements in the frame-by-frame reading results between two investigators, which were resolved after the results were reanalyzed and discussed to reach a consensus.

#### Definitions of Hyoid Bone Movement Events Observed During VFSS

We defined three events of hyoid bone movement (V1, V2, and V3) during VFSS [4] along with three thumb press signals on the handheld FSR sensor (VT1, VT2, and VT3). Accordingly, the onset times of six events (Table 2) and five periods (Table 3) were defined and analyzed.

**Table 2:** Definition of six VFSS events

VF 6 events	Physiological activity of the hyoid bone
1. VT1	First thumb press on handheld FSR sensor
2. VT2	Second thumb press on handheld FSR sensor
3. V1	Onset of movement of the hyoid bone (begin to move from initial position)
4. V2	Onset of stationary phase of the hyoid bone (upmost position)
5. V3	End of offset of movement of the hyoid bone (back to initial position)
6. VT3	Third thumb press on handheld FSR sensor

**Table 3:** Definition of 5 time periods (duration)

#1	VT1–VT2	First thumb press handheld FSR sensor to second thumb press handheld FSR sensor
#2	VT1–V1	First thumb press handheld FSR sensor to the onset of movement of the hyoid bone
#3	VT1–V2	First thumb press handheld FSR sensor to the onset of stationary phase of the hyoid bone
#4	VT1–V3	First thumb press handheld FSR sensor to the offset of stationary phase of the hyoid bone
#5	VT1–VT3	First thumb press handheld FSR sensor to third thumb press handheld FSR sensor

### Defining the waveforms of thyroid cartilage excursion that detected and recorded using FSR sensor and the waveforms of thumb pressed on the handheld FSR sensor (Figs. 2 and 3)

FSR sensors were used to detect thyroid cartilage excursion during swallowing. A W-shaped waveform with two “V”s was recorded. F1 is the onset time of W, F2 is the turning point of W, and F3 is the offset time. A semiautomatic analysis software based on LabView was used, which detected and analyzed all three swallowing-related signals (nasal airflow, submental sEMG, and thyroid cartilage excursion) locked in the same frame of three different traces [26] and combined with the three signal trace from thumb pressing on FSR sensor. The three individual onset times of thumb pressing on the handheld FSR sensor were represented as T1, T2, and T3, comprising two pressings (T1 and T2) before swallowing action and one (T3) after swallowing (Figs. 2 and 3). The onsets of T1, T2, and T3 are defined and marked in Fig. 2; these were overlapped in the uppermost trace panel of submental sEMG. After the signals were defined and analyzed using LabView (Fig. 2), the data were exported to an MS Excel file for subsequent statistical analysis. This automatic output to an MS Excel file saved time and circumvented human error in the process of data entry.

### Data Analysis

The point VT1 in VFSS and FT1 by FSR sensor were defined as timepoint zero (Fig. 3). Consecutively, the timepoints of VT2, VT3, V1, V2, and V3 corresponded to the FT2, FT3, F1, F2, and F3, respectively. These were all critical onset times for specially defined events in both swallowing study images.

### Statistical Analysis

Statistical analyses were performed using SPSS statistical package (version 12.0; SPSS, Inc., Chicago, IL, USS). To determine the correlation between the hyoid motion during VFSS and thyroid cartilage excursion by using FSR in a noninvasive system, Pearson’s correlation coefficients (ϒs) were calculated. Statistical significance between the parameters of the two study systems was taken as a *p*-value of < 0.01 for a two-tailed T test.

## Results

The temporal parameters hyoid motion detected during VFSS and thyroid cartilage excursion detected using FSR sensors demonstrated highly significant correlation (*R* = 0.838–0.990, all *p* < 0.001; data not shown). No significant differences were noted in the temporal parameters between the two tests for each corresponding time duration (VT1–T2 vs FT1–F2, VT1–T3 vs FT1–T3, VT1–V2 vs FT1–F2, and VT1–V3 vs FT1–F3), except for the onset of hyoid motion (VT1–V1) and of thyroid cartilage excursion (FT1–F1), which demonstrated a significant difference (*p* = 0.004; data not shown).

We next analyzed the data in separate groups of men and women. The defined temporal parameters for analysis between VFSS and noninvasive systems demonstrated strong correlations in men (*R* = 0.953–0.999; all *p* < 0.001). In women, the correlation was strong for all (*R* = 0.813–0.982; all *p* < 0.001), except VT1–V1 and FT1–F1, which displayed moderate correlation (*R* = 0.648; *p* = 0.001; Table 4). In men, the five corresponding time durations between the two tests was statistically nonsignificant, whereas only VT1–V1 and FT1–F1 displayed significant differences in women (*p* = 0.001; Table 5).

**Table 4:** Pearson correlation of temporal parameters between VFSS and noninvasive swallowing assessment using FSR sensor in young volunteers without dysphagia [*n* = 29 (Men: 15, Women: 14)]

	Male (No:15)		Female (No:14)	
	R	*P* value	R	*P*-value
VT1-T2 vs FT1-T2	0.998	< 0.001*	0.982	< 0.001*
VT1-T3 vs FT1-T3	0.999	< 0.001*	0.826	< 0.001*
VT1-V1 vs FT1-F1	0.960	< 0.001*	0.648	< 0.001*
VT1-V2 vs FT1-F2	0.984	< 0.001*	0.955	< 0.001*
VT1-V3 vs FT1-F3	0.953	< 0.001*	0.813	< 0.001*

*P-value < 0.01

**Table 5:** Test comparison of temporal parameters of swallowing signals between VFSS and FSR sensor in young volunteers without dysphagia (*n* = 29)

Male (No:15)			Female (No:14)		
VFSS	FSR	*P*-value	VFSS	FSR	*P*-value
VT1-T2 1.153 ± 0.394 (S)	FT1-T2 1.153 ± 0.392 (S)	0.995	VT1-T2 0.996 ± 0.439 (S)	FT1-T2 1.008 ± 0.428 (S)	0.884
VT1-T3 4.494 ± 1.075 (S)	FT1-T3 4.494 ± 1.073 (S)	0.997	VT1-T3 4.678 ± 0.830 (S)	FT1-T3 4.783 ± 0.875 (S)	0.521
VT1-V1 2.375 ± 0.791 (S)	FT1-F1 2.553 ± 0.789 (S)	0.267	VT1-V1 2.241 ± 0.580 (S)	FT1-F1 2.630 ± 0.575 (S)	0.001*
VT1-V2 2.834 ± 0.782 (S)	FT1-F2 2.971 ± 0.731 (S)	0.451	VT1-V2 2.916 ± 0.429 (S)	FT1-F2 3.128 ± 0.458 (S)	0.032
FT1-V3 3.697 ± 0.820 (S)	FT1-F3 3.808 ± 0.809 (S)	0.501	FT1-V3 3.717 ± 0.595 (S)	FT1-F3 3.910 ± 0.584 (S)	0.099

*S* second

*p < 0.01

However, a limitation of this study is that the time scales of the two swallowing measurement systems were different. The time interval by the noninvasive system was 0.001 s, but the interval for VFSS was 0.033 s. This scale difference may have affected the correlation analysis results between the two measurement systems.

## Discussion

This study was the first to analyze concurrent noninvasive sensors and VFSS recordings of swallowing signals. A previous study investigating swallowing signals using a bend sensor to detect thyroid cartilage motion and VFSS to monitor hyoid motion and demonstrated a positive correlation in their results; however, in this study, swallowing signals detection using bend sensors and VFSS was not simultaneously, and it recruited only six young healthy men without dysphagia were recruited, but no women [4]. Another study synchronized the piezoelectric pulse transducer (PPT) waveforms to VFSS recordings of pharyngeal swallowing events in three healthy men [27]. Therefore, thus far, data on the correlation between surface sensors waveforms of swallowing and VFSS as well as a lack of synchronized and simultaneous recordings of two swallowing study methods have been limited.

In the current study, we recruited more normal healthy young volunteers—both men and women—and determined that both men and women displayed strong correlation of temporal parameters between the two swallowing test systems (the thyroid cartilage excursion using FSR through the noninvasive method and the hyoid motion during VFSS). There were no significant differences in temporal parameters between the two systems, except for that in one temporal parameter (VT1–V1 to FT1–F1) in women that displayed a statistically significant difference but a high correlation. Unlike in the study on the correlation of bend sensor and VFSS [4], we simultaneously recorded swallowing events with two systems.

Findings on the effects of gender on swallowing thus far have been inconclusive: some have indicated differences in swallowing parameters between genders, whereas have not [28–33]. These inconclusive findings complicate the data analysis and the interpretation of our results, necessitating more data for confirmation. Our previous study recruited 19 young normal volunteers (9 men and 10 women) to compare the difference of two placement levels of piezoelectric sensors for detecting thyroid cartilage excursion as well as understanding the sex differences. Piezoelectric sensor recordings and surface electromyography were collected during the swallowing process. The sensor placement at the thyroid cartilage level afforded a greater amplitude of laryngeal excursion than did that at the cricothyroidotomy level. Furthermore, in women, oropharyngeal phase-related parameters differed significantly more at the thyroid cartilage level than at the cricothyroidotomy level [9]. In this study, the FSR sensor to monitor the thyroid cartilage excursion was placed at the thyroid cartilage level, which may account in part for our finding that women did not display a strong correlation between the FSR and VFSS results.

When simultaneously recording the swallowing signals of a noninvasive FSR sensor swallowing study system and VFSS, some technical issues need to be solved using hardware and software. To synchronize the two systems, a handheld stick with an FSR sensor, connected to one of the multiple MP150 system channels, was used in this study. Our handheld stick design could be used in future swallowing studies to create timepoint references to aid in synchronizing FSR with VFSS or other recording methods.

This noninvasive swallowing study method using FSR sensors to detect thyroid cartilage excursion of swallowing is reliable and efficient at indirectly detecting the temporal parameters of oropharyngeal swallowing, which has uses in clinical application and future studies. This system has multiple channels to simultaneously record, and time-lock, swallowing-related physiological signals including submental sEMG, nasal airflow, and uncalibrated measurement of global laryngeal excursion, which is adequate to detect a swallowing event. Swallowing events were analyzed but the detailed parameters were not. The reliability of the detection of swallowing events has been demonstrated, and thus, this noninvasive system can be used to analyze piecemeal deglutition and dysphagia limits [26]. These findings are similar to those of a previous report that PPT waveforms corresponded to pharyngeal phase swallowing under VFSS [27]. Moreover, with nasal airflow monitoring, our noninvasive system can be used in studies of swallowing and respiration coordination which include swallowing respiratory pause and pre- and post-swallowing respiratory phase patterns. Furthermore, a stepwise volume increase in swallowing water bolus protocol would not only be beneficial for safety but also could be used to detect the piecemeal deglutition that appears after a water bolus of < 20 mL administered [1–3, 7, 14]. Another principal outcome is that the protocol and analysis methods in this noninvasive swallowing and respiration study system can be used to detect subtle, subclinical [1], and early [2] mild dysphagia. Furthermore, this system can be used for the older people [7], during follow-up [3], and in treatment effectiveness studies [14]. Regarding improvements and applications, advancements in hardware and software are crucial. We have made our system ambulatory and plan to create a portable version. For the pediatric population, we plan to find small surface sensors to detect thyroid cartilage excursion during swallowing.

Swallowing study objective instruments all have various advantages and limitations. The numerous facets of swallowing function studies include temporal parameters [27, 29], dynamic biomechanics [19, 34], and pressure measurement [35]. One study tool cannot be substituted for another, and using multiple study tools can assist, supplement, or compensate for the other swallowing study tools [35, 36]. Therefore, using different swallowing study instruments to examine various facets of swallowing could prove valuable with regards to clinical applications and research. Noninvasive swallowing detection systems is an objective tool that is gaining attention because it demonstrates promising results and thus should be investigated and developed further. The improvement and diversification of the noninvasive swallowing detection system is crucial.

The noninvasive swallowing study without radiation exposure is suitable for posttherapy monitoring of the immediate effects. Furthermore, the advantages of noninvasive swallowing study systems include the possibility of a larger quantity of swallowing events data that can be collected for a cross-section study, a larger number of repeated measurements over a short period in follow-up studies, and studies for swallowing data collection over a long follow-up duration. It is also useful in long-term objective follow-up studies. Another valuable potential application of a noninvasive swallowing system is in feedback training [37, 38]. Our system had already been programmed to monitor nasal airflow for respiration coordination during swallowing, which can be used in visual feedback training with signals displayed on screen.

Noninvasive systems are, however, not without limitations. The noninvasive system cannot record through the oropharyngeal anatomic structures and hyoid displacement, UES relaxation, bolus passage, penetration, aspiration, and residuals after swallowing were recorded during VFSS [39]. These findings have clinical applicability in making decisions for swallowing training programs and deciding whether to remove nasogastric tubes. Noninvasive swallowing study cannot replace VFSS. The noninvasive swallowing study was developed to complete the information obtained from analysis with VFSS [10, 35, 36, 40–47].

Developing automatic analysis software systems to analyze the dynamic images and temporal parameters of swallowing would be valuable in both VFSS and noninvasive swallowing study systems. Using artificial intelligence to solve this semiautomatic analysis problem may lead noninvasive swallowing studies to another frontier.

## Conclusions

This is the first study to concurrently record noninvasive swallowing signals and perform VFSS. We provided a new method for researchers to conduct correlation studies between noninvasive swallowing studies and VFSS.

Our results demonstrated a strong correlation in the temporal parameters of swallowing between noninvasive surface sensors and VFSS. This is crucial for further usage of this noninvasive swallowing study tool in studies and clinically.
